# Limits and potential of targeted sequencing analysis of liquid biopsy in patients with lung and colon carcinoma

**DOI:** 10.18632/oncotarget.10704

**Published:** 2016-07-19

**Authors:** Anna Maria Rachiglio, Riziero Esposito Abate, Alessandra Sacco, Raffaella Pasquale, Francesca Fenizia, Matilde Lambiase, Alessandro Morabito, Agnese Montanino, Gaetano Rocco, Carmen Romano, Anna Nappi, Rosario Vincenzo Iaffaioli, Fabiana Tatangelo, Gerardo Botti, Fortunato Ciardiello, Monica R. Maiello, Antonella De Luca, Nicola Normanno

**Affiliations:** ^1^ Laboratory of Pharmacogenomics, CROM-Istituto Nazionale Tumori “Fondazione G. Pascale”-IRCCS, Naples, Italy; ^2^ Thoraco-Pulmonary, Medical Oncology, Istituto Nazionale Tumori “Fondazione G. Pascale”-IRCCS, Naples, Italy; ^3^ Thoracic Surgery, Istituto Nazionale Tumori “Fondazione G. Pascale”-IRCCS, Naples, Italy; ^4^ Gastro-Intestinal Medical Oncology, Istituto Nazionale Tumori “Fondazione G. Pascale”-IRCCS, Naples, Italy; ^5^ Surgical Pathology Unit, Istituto Nazionale Tumori “Fondazione G. Pascale”-IRCCS, Naples, Italy; ^6^ Department of Clinical and Experimental Medicine ‘F. Magrassi’-Medical Oncology, Seconda Università degli Studi di Napoli, Napoli, Italy; ^7^ Cell Biology and Biotherapy Unit, Istituto Nazionale Tumori “Fondazione G. Pascale”-IRCCS, Naples, Italy

**Keywords:** targeted sequencing, colon cancer, lung cancer, liquid biopsy, driver mutations

## Abstract

The circulating free tumor DNA (ctDNA) represents an alternative, minimally invasive source of tumor DNA for molecular profiling. Targeted sequencing with next generation sequencing (NGS) can assess hundred mutations starting from a low DNA input. We performed NGS analysis of ctDNA from 44 patients with metastatic non-small-cell lung carcinoma (NSCLC) and 35 patients with metastatic colorectal carcinoma (CRC). NGS detected EGFR mutations in 17/22 plasma samples from EGFR-mutant NSCLC patients (sensitivity 77.3%). The concordance rate between tissue and plasma in NSCLC was much lower for other mutations such as KRAS that, based on the allelic frequency and the fraction of neoplastic cells, were likely to be sub-clonal. NGS also identified EGFR mutations in plasma samples from two patients with EGFR wild type tumor tissue. Both mutations were confirmed by droplet digital PCR (ddPCR) in both plasma and tissue samples. In CRC, the sensitivity of the NGS plasma analysis for RAS mutations was 100% (6/6) in patients that had not resection of the primary tumor before blood drawing, and 46.2% (6/13) in patients with primary tumor resected before enrollment. Our study showed that NGS is a suitable method for plasma testing. However, its clinical sensitivity is significantly affected by the presence of the primary tumor and by the heterogeneity of driver mutations.

## INTRODUCTION

The identification of driver mutations in a large fraction of patients with solid tumors and the availability of drugs that are able to block the activation of specific signaling pathways is boosting the development of a novel therapeutic approach defined as genomic-driven oncology [1]. This definition might be considered a synonymous of personalized medicine or precision medicine, in which treatment of the single cancer patient is driven by a specific genetic alteration of the tumor, i.e. a specific biomarker.

There are several issues that limit the development of this new therapeutic strategy. First, in some patients tissue might not be available for molecular testing. Indeed, it has been estimated that approximately 25% of lung carcinoma patients have no tissue available for EGFR testing after completion of histologic assessment. In addition, evidence suggests that tumors are highly heterogeneous [2, 3]. Therefore, a single tumor biopsy might not allow to represent the comprehensive genetic landscape of the disease. Finally, the molecular profile of the tumor significantly changes following treatment with targeted agents [4]. As a consequence, molecular monitoring of the disease over the time is necessary in order to identify mechanisms of resistance and to adapt the therapy to the new molecular landscape.

Several of these issues can be overcome by liquid biopsy. With this term we refer to the possibility to perform the molecular profile of the tumor by analyzing the circulating free tumor DNA (ctDNA) that can be isolated from peripheral blood [5]. Several studies have shown that it is possible to detect somatic mutations in ctDNA and this approach is entering in the clinical practice. In particular, EGFR testing on liquid biopsy for patients with non-small-cell lung carcinoma (NSCLC) has been approved in Europe based on the results of the IFUM trial that showed an acceptable sensitivity and specificity of this approach [6]. In this study, liquid biopsies were assessed with the Therascreen kit, and a sensitivity of 65.7% and a specificity of 99.8% were observed [7]. Studies are ongoing to validate liquid biopsy for the detection of RAS mutations in patients with metastatic colorectal carcinoma (CRC).

The majority of the studies published on liquid biopsy have used methods that can assess few mutations per analysis. However, the number of possible targets for therapeutic intervention is exponentially growing. For example, potential driver alterations have been identified in up to 75% of NSCLC [8]. Similarly, the number of actionable mutations identified in CRC significantly increased in the past few years [9]. Assessment of a large number of biomarkers requires the use of novel techniques that allow multiplex biomarker testing. In this regard, targeted sequencing with next generation sequencing (NGS) has the advantage to assess hundred mutations starting from a low DNA input and, therefore, to provide a comprehensive molecular portrait [10]. Some studies have shown that targeted resequencing is a suitable technique for ctDNA analysis [11–14]. However, most of these studies have focused on limited cohorts of patients and/or have employed laboratory-developed techniques that are not immediately transferable to the clinic.

In this manuscript we describe the results of testing ctDNA with an NGS-based panel that has been approved for *in vitro* clinical diagnostics in colon and lung cancer patients (CE-IVD). The potential and the limits of this approach are described in order to provide information on the possible use of this panel in a clinical scenario.

## RESULTS

### Patients characteristics

Forty-four patients with metastatic NSCLC and 35 metastatic CRC patients were included in the study. Clinical and pathological features of patients are shown in Table 1. NSCLC patients were previously screened for EGFR mutations whereas CRC patients were tested for KRAS and NRAS mutations by using standard diagnostic methods.

**Table 1:** Patients' characteristics

Patients	Lung Cancer	Colon Cancer
**n°**	44	35
**Age (mean)**	62,5	62,5
**Sex**		
Male	23	20
Female	21	15
**Stage**		
III	1	–
IV	43	35
**Primary tumor**		
resected	10	19
not resected	34	16
**Metastasis**		
M1a	5	–
M1b	38	–
**N° metastatic sites**		
1	12	15
> 2	31	20
**EGFR status**		
mutant	22	–
wild type	22	–
**RAS status**		
mutant	–	19
wild type	–	16

### Targeted sequencing of tissue and plasma samples from EGFR mutant NSCLC patients

**Figure 1:**
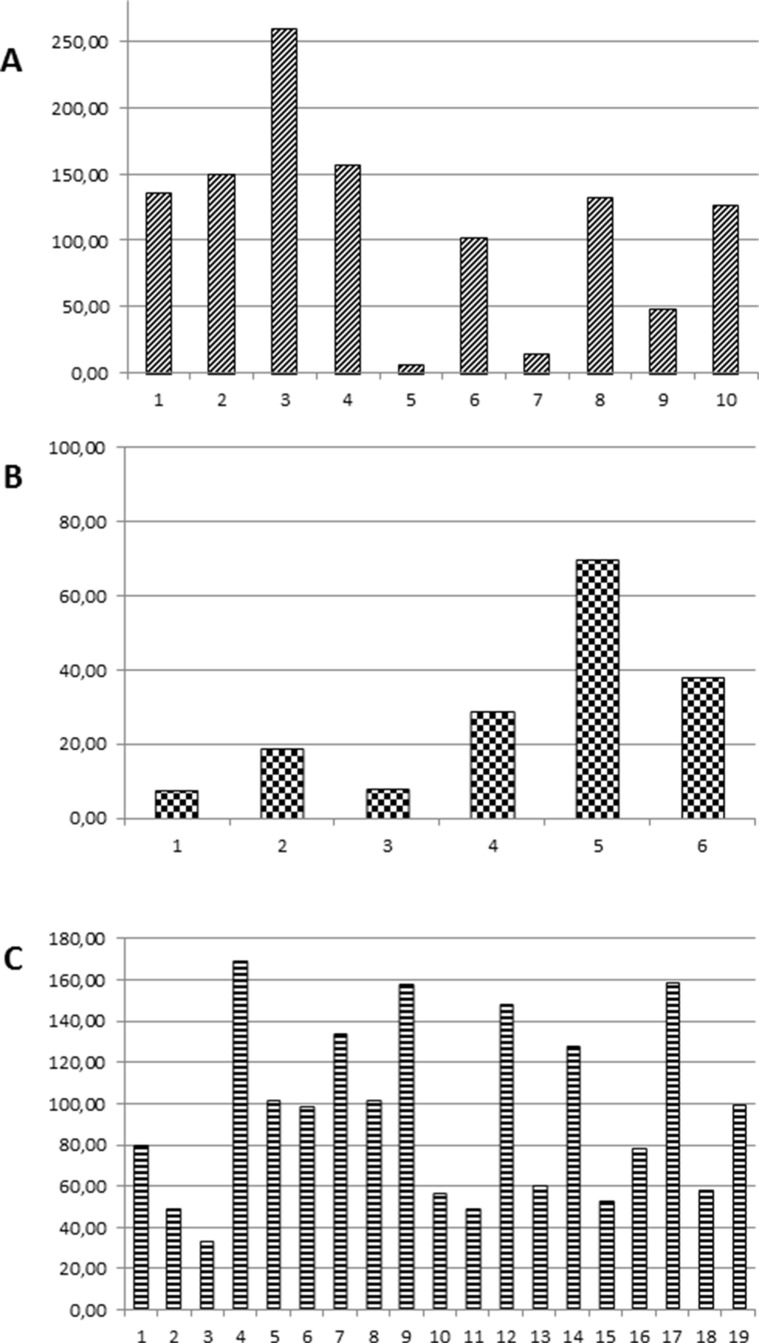
Heterogeneity Score values of: (A) EGFR mutations in NSCLC; (B) KRAS mutations in NSCLC; (C) KRAS and NRAS mutations in CRC

Analysis of tissue or cytology samples by NGS confirmed the EGFR mutation in all cases (n.22) that were previously assessed as EGFR mutant (Supplementary Table 1). The allelic frequency of the EGFR mutation ranged between 2.6% and 87.7%, with mean and median values of 37.44 and 47.9, respectively. We previously described the Heterogeneity Score as a tool to normalize the allelic frequency of a mutation for the fraction of neoplastic cells in order to better represent tumor heterogeneity [15]. The fraction of neoplastic cells was estimated by two independent observers in 10 histological samples (Supplementary Table 1). The heterogeneity score for EGFR mutations ranged between 6.5 and 259.2, with a mean value of 113.1 and a median value of 129, thus suggesting that in the majority of cases the EGFR mutation was clonal (Figure 1A).

NGS sequencing detected EGFR mutations in 17/22 plasma samples from EGFR mutant NSCLC patients, thus resulting in a sensitivity of 77.3% (Table 2). All cases that were positive by NGS also resulted EGFR mutant according to analysis with the Therascreen kit with the exception of case L13 that was negative on plasma for the Therascreen (Supplementary Table 1). The 5 cases false negative on plasma were all carrying exon 19 deletions. In one case the NGS and the Therascreen revealed in the plasma the EGFR exon 19 deletion but not the p.T790M mutation that was identified in the tumor tissue (case L2, Supplementary Table 1). Analysis of the plasma sample with droplet digital PCR (ddPCR) confirmed the presence of the p.T790M mutation at an allelic frequency of 0.27%. Material was available to perform ddPCR analysis in 3/5 cases that were positive on tumor and negative on plasma by NGS. Two cases were found positive for exon 19 deletions at ddPCR analysis with frequency of mutant alleles of 0.5% and 5.3%, respectively. The exon 19 variant identified by ddPCR at a frequency of 5.3% was a relatively rare complex deletion (p.E746_S752>V).

**Table 2:** NGS analysis in tissue/cytological and plasma samples from NSCLC patients

Tumor EGFR status	Plasma EGFR status		
	Wild Type	Mutant
**Wild Type**	20	2
**Mutant**	5	17

Twenty patients received first line treatment with an EGFR tyrosine kinase inhibitor (TKI), and 16 of these patients had also a liquid biopsy positive for EGFR mutations. Patients with tissue positive for EGFR mutations and those with both tissue and liquid biopsy positive for EGFR mutations showed similar response rate (65% vs 68.8%), disease control rate (80% vs 81.2%) and median progression free survival (9 months vs 8.7 months) following TKI treatment.

Tissue and plasma analysis with NGS revealed variants in genes additional to the EGFR in selected cases (Supplementary Table 1). The agreement between tissue and plasma for mutations other than EGFR was lower as compared with EGFR variants (Supplementary Table 1). In particular, variants present in the tumor tissue were in several cases not detected in plasma, whereas plasma analysis revealed variants that were not found in the tumor specimens. In selected cases where material and assays were available, it was possible to confirm the new variants identified in plasma, thus confirming that they are not sequence artifact but rather reflect an higher heterogeneity of EGFR mutant tumors as compared to what revealed by tumor tissue analyses.

### Targeted sequencing of tissue and plasma samples from EGFR wild type NSCLC patients

NGS analysis of tissue/cytology samples from 22 NSCLC patients that were previously assessed as EGFR wild type according to standard clinical diagnostic testing did not reveal EGFR mutations. However, analysis of plasma samples with NGS identified two EGFR mutations in patients with EGFR wild type tumor tissue (cases L29 and L33; Tables 2 and 3; Supplementary Table 2). Both mutations were confirmed by ddPCR. In addition, analysis with ddPCR of the genomic DNA derived from tumor specimens confirmed the presence of the EGFR mutations, although at allelic frequencies that are below the threshold of sensitivity of both NGS and the Therascreen EGFR kit that was employed for routine diagnostics (0.23% and 0.76%; Supplementary Table 2).

Mutations in KRAS, TP53, CTNNB1, MET, FBXW7, and PIK3CA were also identified in tissue and/or plasma from EGFR wild type patients. The concordance rate between tissue and plasma was much lower as compared with EGFR mutations (Table 3). For example, KRAS mutations were found in 8/22 tumor samples (36.4%). However, the same KRAS mutation was identified only in 1/8 plasma samples, whereas in two additional patients a KRAS mutation was found in the plasma but not in the tumor tissue. In one of these cases it was possible to analyze the tumor tissue by ddPCR and the analysis confirmed the presence of the KRAS mutation detected in the ctDNA, although at a very low frequency (case L41: KRAS p.G13D 0.03%; Supplementary Table 2).

**Table 3:** Mutations revealed by NGS in EGFR wild type NSCLC patients

	EGFR	KRAS	TP53	CTNNB1	FBXW7	PIK3CA	MET
**L24**							
**L25**							
**L27**							
**L28**							
**L29**							
**L31**							
**L32**							
**L33**							
**L34**							
**L37**							
**L38**							
**L41**							
**L42**							

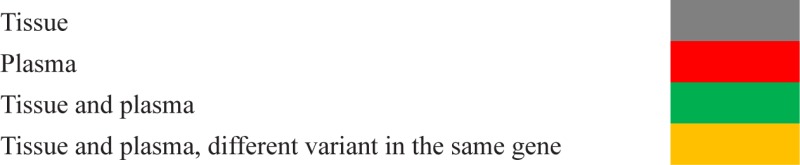

The lower sensitivity for KRAS mutation as compared with EGFR mutations could be due to the different tumor load. Although the majority of both EGFR and KRAS mutant cases were M1b, only 2/22 (9%) EGFR mutant cases had the primary tumor resected versus 4/8 (50%) KRAS mutant patients (Supplementary Tables 1 and 2). However, tumor heterogeneity might play a role in this phenomenon as well. In fact, the allelic frequency of the KRAS mutations in the tumor samples ranged between 2.2% and 79.8%, with mean and median values of 21.58 and 10.65, respectively. When the allelic frequency of the KRAS mutations was normalized for the fraction of neoplastic cells in the six histologic samples, the Heterogeneity Score ranged between 7.33 and 69.33, with a mean value of 28.21 and a median value of 23.62 (Figure 1B), suggesting that in most of the tumor samples of our series the KRAS mutation was sub-clonal. Similar observations were made also for the TP53 mutations (Supplementary Table 2 and data not shown).

### Targeted sequencing of CRC patients' tissue and plasma samples

Targeted sequencing of tumor specimens from 35 patients with metastatic CRC confirmed the RAS mutational status previously assessed with routine diagnostic techniques (Supplementary Table 3). The allelic frequency of the RAS mutant allele ranged between 13.2% and 55.6%, with mean and median values of 30.73 and 27.2, respectively. The Heterogeneity Score of the RAS mutant colorectal carcinoma samples ranged between 33 and 168.8, with a mean value of 95.4 and a median value of 98.5 (Figure 1C), which suggested that in the majority of the tumors the RAS mutations were clonal.

The sensitivity of the NGS plasma analysis was only 63.2%, with 12/19 RAS mutant patients having a plasma RAS mutation (Table 4). Despite NGS identified the same RAS mutation in both tumor and plasma in 6/6 patients that had not resection of the primary tumor before blood drawing, the sensitivity dropped to 46.2% (6/13) in patients that received surgery of the primary before enrollment in the study (*p* = 0.04; Fisher's exact test). Material for ddPCR analysis was available for 5 negative cases. In 3/5 samples ddPCR identified RAS mutations at a frequency ranging between 0.5% and 0.93%. Overall, these data suggest a direct correlation between the presence of the primary tumor, levels of mutant DNA in the plasma samples and success of NGS plasma analysis. In agreement with this hypothesis, target sequencing of tissue samples from 16 RAS wild type patients identified 11 mutations in 9 patients that had no resection of the primary tumor (Supplementary Table 3). Nine out of 11 mutations were also detected by NGS in the plasma samples with a sensitivity of 81.8% (Table 5).

**Table 4:** RAS mutational status of CRC patients according to NGS analysis of tissue and plasma samples

Tumor RAS status	Plasma RAS status			
	Primary Tumor not resected		Primary Tumor resected	
	Wild Type	Mutant	Wild Type	Mutant
**Wild Type**	11	0	5	0
**Mutant**	0	6	7	6

**Table 5:** Mutations revealed by NGS analysis in RAS wild type CRC patients

	TP53	PIK3CA	BRAF	SMAD4	EGFR	FBXW7
C20*						
**C22**						
**C23**						
**C24**						
**C25**						
C26*						
**C27**						
C28*						
**C29**						
**C31**						
**C34**						
**C35**						

* primary tumor resected

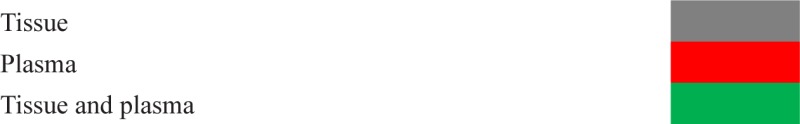

Finally, in order to improve the sensitivity of the assay, a new design of the panel with shorter amplicons was generated. However, this resulted only in a slight increase of the sensitivity. In fact, a RAS mutation was identified in one additional patient (case C9), thus leading to an overall sensitivity of 68.4% (13/19). The new panel also identified a KRAS p.G12V mutation in the plasma from a patient that had a KRAS p.G12C mutation in the tumor tissue (case C2). The p.G12V variant was confirmed by ddPCR in plasma but not in the tumor sample, thus suggesting tumor heterogeneity. In this regard, the heterogeneity score for the KRAS p.G12C mutation in this tumor was < 50, confirming that only a fraction of neoplastic cells were carrying the mutation.

## DISCUSSION

We have previously demonstrated that targeted sequencing is able to identify with high sensitivity and specificity somatic mutations in tumor-derived DNA, even when starting from the limited amount of nucleic acids that can be obtained from cytology samples [16]. Several studies have also investigated the possibility to use targeted sequencing to assess somatic mutations in ctDNA and have reported sensitivities ranging between 58% and 97% [11–14]. However, small cohorts of patients have been analyzed in these studies using different techniques. In this study, we explored the use of a NGS-based panel approved for clinical diagnostics to analyze plasma samples from NSCLC and CRC patients, in order to define the potential and limits of this approach.

The sensitivity of NGS to detect sensitizing EGFR mutations in ctDNA from NSCLC patients was 77.2%, even higher as compared with previous reports that used the Therascreen kit [7, 17]. Importantly, the outcome of patients who received EGFR TKI as first line treatment and who were tumor EGFR mutation positive or tumor and plasma positive was similar, thus confirming that NGS analysis of liquid biopsy is a suitable method for EGFR mutation testing in NSCLC [18]. Several studies have suggested a possible correlation between clinical and pathological characteristics of the disease and the amount of ctDNA that can be isolated from the plasma of cancer patients [19, 20]. In this respect, a previous study showed that liquid biopsy for the p.T790M mutation had an higher chance of success in NSCLC patients with extra-thoracic metastatic disease (M1b) as compared to patients with disease confined to the thoracic cavity (M1a/M0) [21]. By chance, in our study 18/22 (81.8%) patients with EGFR mutations had extra-toracic disease and this might have determined the high sensitivity of the NGS-assay.

Although NGS was quite sensitive to detect EGFR mutations in plasma, the agreement between tissue and plasma for additional coexisting mutations identified in EGFR mutant NSCLC was low, thus preventing the possibility to have a comprehensive picture of tumor heterogeneity using liquid biopsy. This might be clinically relevant, because the presence of additional coexisting mutations in EGFR mutant NSCLC has been reported to be associated with a worse outcome in EGFR mutant patients treated with EGFR TKI [22], although other studies did not find such correlation [23]. Interestingly, in our series 3/4 EGFR mutant patients with progressive disease after EGFR TKI treatment had coexisting mutations in KRAS or BRAF in the primary tumor, in two cases at an allelic frequency higher than EGFR mutations. In one case the liquid biopsy was negative; in the other two cases only the EGFR mutation was detected in the plasma samples.

The correlation between the presence of the primary tumor and the success of liquid biopsy analysis was evident in CRC patients. In fact, the sensitivity of the NGS panel was much higher in those patients that had still the primary tumor at the time of blood drawing as compared with patients that had the primary tumor resected. In this latter cohort of patients, the sensitivity was < 50% which is not acceptable in clinical practice. In fact, a false negative result might lead to treatment of a RAS mutant patient with anti-EGFR monoclonal antibodies. In this regard, different studies have demonstrated that EGFR targeting drugs have a detrimental effect on survival of CRC patients when combined with oxaliplatin-containing regimens [24, 25]. Therefore, an high sensitivity and specificity is required for RAS testing in CRC. The sensitivity of the NGS panel for KRAS mutations was quite low also in NSCLC. In this respect, the high rate of NSCLC patients with primary tumor resected among KRAS mutant cases might have accounted for the low detection rate of KRAS mutation in plasma. Interestingly, the NGS panel had an high sensitivity to detect mutations other than RAS in CRC patients with non-resected primary tumor. However, the possibility to detect these mutations is also likely to be affected by the extent of the tumor burden.

The limit of detection of targeted sequencing is approximately 1%. Methods with a lower limit of detection might result in an higher clinical sensitivity [26]. Indeed, ddPCR could identify EGFR or RAS mutations in 6/10 false negative cases by NGS, usually at an allelic frequency <1%. However, it must be emphasized that some samples were negative also at ddPCR analysis, thus suggesting that a fraction of cancer patients might not have a sufficient level of ctDNA for mutational analysis. Because of the possible implications of false negative results, this observation highlights the importance to identify clinical characteristics that might help to select patients that are likely to have sufficient levels of ctDNA. Interestingly, a recent report showed a correlation between the frequency of EGFR mutations in ctDNA and outcome in NSCLC patients, thus suggesting that the presence of circulating EGFR mutant DNA might have biological and clinical implications [27].

An important hypothesis deriving from our data is that tumor heterogeneity might also affect the possibility to reliably identify somatic mutations in ctDNA. Indeed, the sensitivity of NGS to detect KRAS mutations in EGFR wild type cases was much lower as compared with the detection of EGFR mutations. By using the Heterogeneity Score, we found that KRAS mutations were likely to be subclonal in the majority of NSCLC cases that we analyzed, whereas EGFR mutations in NSCLC seem to be in most cases clonal or at least represented in the majority of neoplastic cells. Although these results might have been affected by the possible amplification of the EGFR gene that occurs in a fraction of EGFR mutant NSCLC, analysis of the NGS data seem to exclude that copy number variation occurred in the samples that we assessed for the Heterogeneity Score. Previous studies have demonstrated that NSCLC adenocarcinoma are often formed by different clones of neoplastic cells [3]. In addition, KRAS mutations, as well as other driver mutations including EGFR variants, have been shown to be either clonal or sub-clonal in NSCLC adenocarcinoma [2]. It is possible that our findings in KRAS mutant NSCLC have been influenced by the small number of samples analyzed and by the high rate of resection of the primary tumor. Nevertheless, further studies are necessary to elucidate the clonal nature of KRAS mutations in NSCLC and its implications for the liquid biopsy.

An additional consequence of tumor heterogeneity is that some mutations might be identified in plasma but not in the primary tumor tissue. This is not surprising, because increasing evidence suggests that often genetic heterogeneity occurs between lung primaries and metastases [28]. In our study, two patients with EGFR wild type tumors had EGFR mutations in plasma. Previous studies of EGFR mutation detection in plasma have shown similar results, with some plasma samples resulting positive in patients with the tumor tissue negative for EGFR mutations [29]. We confirmed the identified mutations by ddPCR in both tissue and plasma in both cases, thus demostrating the specificity of the NGS plasma analysis. However, these data open questions on the clinical interpretation of these findings because EGFR mutations were at a very low frequency in the tumor tissue. Although no quantitative threshold of EGFR mutation has been established to identify NSCLC patients that might benefit of treatment with EGFR TKIs, previous studies have suggested a correlation between the “amount” of EGFR mutation and the extent of the response to these agents [22, 30, 31]. Similarly, no quantitative threshold has been identified for biomarkers associated with resistance to targeted agents, such as RAS mutations in CRC [32]. Nevertheless, we must recognize that driver mutations at a very low allelic frequency might not have a relevant role in tumor growth and, therefore, might not be predictive of response to treatment. The main limit of the analysis of ctDNA is that the frequency of the mutation detected in plasma is affected by several variables, including the heterogeneity of the tumor and the possible release of wild type DNA by non-transformed tissues surrounding the tumor as well as by leukocytes. Because we have no possibility at this time to normalize the tumor DNA for the DNA derived from non-transformed cells, it is not possible to estimate the frequency of a mutation in the tumor tissue based on its frequency in the liquid biopsy.

In conclusion, our study showed that NGS is a suitable method for plasma testing. However, the clinical sensitivity of this method was significantly limited by the presence of the primary tumor and by tumor heterogeneity. Techniques with higher analytical sensitivity might reduce the false negative rate, although the identification of variants at low allelic frequency opens questions that need to be addressed in clinical trials.

## MATERIALS AND METHODS

### Tissue and plasma samples

The study was approved by the Ethical Committee of the Pascale Institute: protocol n. 16/14 OSS and protocol n. 8/14 OSS. Consecutive cases fulfilling the inclusion criteria (availability of both plasma and tissue samples for NGS analysis; informed consent) were included in the analysis. Plasma samples were obtained at diagnosis, prior to any systemic treatment. Patients with metastatic NSCLC received EGFR testing on tissue as part of their initial workout. EGFR tissue testing was performed with the Therascreen EGFR RGQ PCR Kit (Qiagen, Milan, Italy) according to manufacturer's instructions. Patients with metastatic CRC received RAS testing on tissue as part of their routine diagnostic analysis. RAS mutational status of tissue samples was previously determined with the Therascreen KRAS and NRAS Pyro kit (Qiagen), according to manufacturer's instructions.

### DNA extraction from plasma samples

Blood samples were collected into 10.0 mL BD Vacutainer® plastic tubes containing EDTA (BD Diagnostics, Milan, Italy). The plasma fraction was separated from the blood cells by two consecutive rounds of centrifugation for 10 min at 4°C, at 1600g and at 3000g, respectively. The collected plasma was aliquoted and stored at −80°C until use. ctDNA was extracted from 2 ml of plasma using the QIAamp Circulating Nucleic Acid Kit (Qiagen) according to manufacturers' instructions. The ctDNA quantity was assessed with the dsDNA HS assay kit by the Qubit 2.0 Fluorometer (Invitrogen, Monza, Italy).

### NGS analysis

Tumor and plasma samples were analyzed with the Oncomine Solid Tumour DNA kit (Life Technologies, Monza, Italy) using the Ion Torrent semiconductor sequencing. The panel analyses hotspot and targeted regions of the following 22 genes implicated in colon and lung cancers: ALK, EGFR, ERBB2, ERBB4, FGFR1, FGFR2, FGFR3, MET, DDR2, KRAS, PIK3CA, BRAF, AKT1, PTEN, NRAS, MAP2K1, STK11, NOTCH1, CTNNB1, SMAD4, FBXW7, TP53. The Oncomine Solid Tumour DNA kit is a single pool panel that uses 92 amplicons (average amplicon length 115 bp) for re-sequencing and analysis. Libraries were prepared starting from 10 ng of genomic DNA or ctDNA (measured using the Qubit fluorometer in combination with the Qubit dsDNA HS assay kit) according to the manufacturer's instructions. The amplified libraries were analysed on the Agilent^®^ 2100 Bioanalyzer instrument with the Agilent High Sensitivity DNA kit (Agilent Technologies, Milan, Italy). 100 pM of each library was multiplexed and clonally amplified on Ion sphere particles (ISPs) by emulsion PCR performed on the Ion One Touch 2 instrument with the Ion PGM template OT2 200 kit (Life technologies) according to the manufacturer's instructions. Quality control was performed using the Ion Sphere Quality Control kit (Life Technologies). Finally, the template ISPs were enriched, loaded on an Ion 316 chip and sequenced on a PGM sequencer with the Ion PGM™ sequencing 200 kit v2 according to the manufacturer's instructions. The raw data were analyzed using the torrent suite software v4.6 (Life Technologies) with a pre-established workflow. Mutations were detected using the Ion Reporter Software v4.6 with low stringency settings (Life Technologies). Threshold for mutation detection was set at 2%. In the variant list obtained, each mutation was verified in the Integrative genome viewer (IGV) from the Broad Institute (http://www.broadinstitute.org/igv/). Plasma samples from CRC patients were also analysed using a modified panel with average amplicon length of 85bp, using the same workflow.

### EGFR mutational analysis of ctDNA with the Therascreen Kit

The EGFR status of ctDNA obtained from NSCLC patients was determined by a modified procedure of the Therascreen EGFR RGQ PCR Kit that has been previously described [17]. Briefly, reactions were performed on the Rotor-Gene Q real-time PCR cycler (Qiagen). Run conditions were modified carrying out 50 cycles of PCR. Data were analysed with the Rotor-Gene Q Series Software (Qiagen).

### Droplet digital PCR analysis

Mutant allele frequency was measured using the QX200 Droplet Digital PCR (ddPCR) System (Bio-Rad, Milan, Italy) in accordance with the manufacturer's instructions. ddPCR reaction mixtures contained a final concentration of 250 nM for each of the probes, 450 nM for the forward and reverse primers, 1x ddPCRTM Supermix for Probes (No dUTP) (Bio-Rad) and 15 ng of genomic DNA or cftDNA in a final volume of 20 μl. The entire volume of this ddPCR reaction volume were loaded in appropriate wells of a DG8 cartridge (Bio-Rad) with 70 μl of generator oil (Bio-Rad). Samples are partitioned into approximately 20,000 water-oil emulsion droplets using the QX200 Droplet generator (Bio-Rad). Forty microliters of the water-oil emulsion were used for the ddPCR reaction that was performed with a Veriti Thermal cycler (Life Technologies) under the following conditions: 1 cycle of 95°C for 10 min, 40 cycles of 94°C for 30s and 55°C for 1 min, and 1 cycle of 98°C for 10 min. After thermal cycling, the plates were transferred to a QX200 Droplet reader. The digital PCR data were analyzed using the QuantaSoft analytical software v1.7.4 (Bio-Rad).

### Heterogeneity score

The Heterogeneity Score was calculated as previously described [15]. Briefly, the mutant allelic frequency was normalized for the neoplastic cell content, in order to calculate the frequency of mutant alleles in neoplastic cells; this value was subsequently multiplied by 2. The Heterogeneity Score virtually corresponds to the fraction of neoplastic cells carrying a specific mutation: HS = 100 suggests that all neoplastic cells carry the mutation; HS < 100 indicates that only a fraction of neoplastic cells is mutant; HS > 100 implies copy number variation (CNV), either gain of the mutant allele or loss of the wild type allele.

## SUPPLEMENTARY MATERIAL TABLES








