# Supplementary material for: Limits and potential of targeted sequencing analysis of liquid biopsy in patients with lung and colon carcinoma
Source: Oncotarget. 2016 Jul 19;7(41):66595–605. doi: 10.18632/oncotarget.10704 (PMC5341823; doi:10.18632/oncotarget.10704)
Supplement: Supplementary file 1 [file oncotarget-07-66595-s001.pdf]

# Limits and potential of targeted sequencing analysis of liquid biopsy in patients with lung and colon carcinoma

## Supplementary Materials

**Supplementary Table S1: - EGFR mutant NSCLC.** See Supplementary_Table_S1

**Supplementary Table S2: - EGFR wild type NSCLC.** See Supplementary_Table_S2

**Supplementary Table S3: - RAS mutant CRC.** See Supplementary_Table_S3
