# Supplementary material for: Limits and potential of targeted sequencing analysis of liquid biopsy in patients with lung and colon carcinoma
Source: Oncotarget. 2016 Jul 19;7(41):66595–605. doi: 10.18632/oncotarget.10704 (PMC5341823; doi:10.18632/oncotarget.10704)
Supplement: Supplementary file 2 [file oncotarget-07-66595-s002.docx]

Supplementary Table S1: - EGFR mutant NSCLC

| ID | Sample type | % or N. neoplastic cells | Expected variants | Mutations found by ION | Coverage | Selected mutations analysed by ddPCR | Age | Stage | Primary tumor resected or not resected | Metastasis (M1a, M1b) | N° metastatic sites | Metastatic site |
| --- | --- | --- | --- | --- | --- | --- | --- | --- | --- | --- | --- | --- |
| L1 | Cytology | 200 cells | EGFR: c.2235-2249del15bp (p.E746-A750delELREA) | EGFR: p.E746_A750del (c.2235_2249del15) (48%); ERBB2: p.P780_Y781ins (c.2340_2341insGGCTCCCCA) (22%); CTNNB1: p.S33Y (c.98C>A) (6%); FGFR3: p.A391E (c.1172C>A) (28%) | 1942 |  | 41 | IV | NO | M1b | > 2 | bone, liver, limph nodes, pericardium, pleura, peritoneum |
|  | Plasma |  | EGFR: DEL exon 19 | EGFR: p.E746_A750del (c.2235_2249del15) (36,6%); KRAS: p.G12V (c.35G>T) (13%) | 5230 | KRAS: c.35G>T (p.G12V) |  |  |  |  |  |  |
| L2 | Biopsy | 80% | EGFR: c.2236-2250del15bp (p.E746-A750delELREA); c.2369C>T (p.T790M) | EGFR: p.E746_A750Del15 (c.2236_2250Del15) (54,5%); p.T790M (c.2369C>T) (23,1%); PIK3CA: p.P539R (c.1616C>G) (4,1%); | 2020 |  | 71 | IV | NO | M1b | > 2 | bone, liver, lung, lymph nodes |
|  | Plasma |  | EGFR: DEL exon 19 | EGFR: p.E746_A750Del15 (c.2236_2250Del15) (1,63%); | 4980 | EGFR T790M: T790M (0,27%) |  |  |  |  |  |  |
| L3 | Cytology | 200 cells | EGFR: c.2237-2255>T (p.E746-S752>V) | EGFR: p.E746_S752>V (c.2237_2255>T) (50,8%);TP53: p.R110L (c.329G>T) (57,7%) | 2170 |  | 55 | IV | NO | M1b | > 2 | lung, bone, brain |
|  | Plasma |  | EGFR: DEL exon 19 | − | 4950 | EGFR: Del (0,5%) |  |  |  |  |  |  |
| L4 | Biopsy | 40% | EGFR: c.2573T>G (p.L858R) | EGFR: p.L858R (c.2573T>G) 30%; TP53: p.C135F (c.404G>T) 22% | 1980 |  | 64 | IV | NO | M1b | 2 | lung and bone |
|  | Plasma |  | EGFR: c.2573T>G (p.L858R) | EGFR: p.L858R (c.2573T>G) 80,4%; TP53: p.C135F (c.404G>T) 39,5% | 5010 |  |  |  |  |  |  |  |
| L5 | Cytology | 200 cells | EGFR: DEL exon 19 | EGFR: p.L747_P753>S (c.2240_2257del18) (6,9%); | 2005 |  | 78 | IV | NO | M1b | > 2 | lung, pleura, bone |
|  | Plasma |  | EGFR: DEL exon 19 | EGFR: p.L747_P753>S c.2240_2257del18 (2,2%) | 5170 |  |  |  |  |  |  |  |
| L6 | Biopsy | 50% | EGFR: c.2573T>G (p.L858R) | EGFR: p.L858R (c.2573_2574TG>GT) (64,8%) | 1970 |  | 48 | IIIB | NO | _ | _ |  |
|  | Plasma |  | EGFR: c.2573T>G (p.L858R) | EGFR: p.L858R (c.2573_2574TG>GT) (1,8%) | 4989 |  |  |  |  |  |  |  |
| L7 | Cytology | 300 cells | EGFR: c.2237_2255del19>T (p.E746_S752>V) | EGFR: p.E746_S752>V (c.2237_2255>T) (60,4%); | 2025 |  | 66 | IV | NO | M1b | 2 | lung and brain |
|  | Plasma |  | EGFR: wild type | FBXW7: p.R465C (c.1393C>T) (3,1%) | 5070 | EGFR:Del (5,3%) |  |  |  |  |  |  |
| L8 | Biopsy | 80% | EGFR: c.2235_2249 del 15 (p.E746_A750del) | EGFR: p.E746_A750del (c2235_2249 del 15) (62,8%); | 1986 |  | 51 | IV | NO | M1b | 2 | bone, brain |
|  | Plasma |  | EGFR: DEL exon 19 | EGFR: p.E746_A750del (c2235_2249 del 15) (3,2%); BRAF: p.T599I (c.1796C>T) (3,9%) | 5130 |  |  |  |  |  |  |  |
| L9 | Cytology | 100 cells | EGFR: c.2239-2251>C (p.L747-T751>P) | EGFR: p.L747-T751>P.; (c 2239-2251>C) (47,8%); | 2175 |  | 67 | IV | NO | M1b | > 2 | brain, bone, liver |
|  | Plasma |  | EGFR: DEL exon 19 | EGFR: p.L747-T751>P.; (c 2239-2251>C) (1,84%) | 5162 |  |  |  |  |  |  |  |
| L10 | Cytology | 100 cells | EGFR: c.2239_2256del18 (p.L747_S752del) | EGFR: p.L747_S752del (c.2239_2256del18) (53,2%) | 2356 |  | 70 | IV | NO | M1b | > 2 | bone, liver, lung, brain |
|  | Plasma |  | EGFR: DEL exon 19 | EGFR: p.L747_S752del (c.2239_2256del18) (2,4%); KRAS: p.G13D (c.38G>A) (6,3%) | 5174 | KRAS: p.G13D (c.38G>A) (3,8%) |  |  |  |  |  |  |
| L11 | Biopsy | 80% | EGFR: DEL exon 19 | KRAS: p.G12A (c.35G>C) (10,7%); EGFR: p.E746_A750>DP (c.c.2238_2249>TCC) (2,6%); BRAF: p.V600E (c.1799T>A) (50,2%); | 1996 |  | 71 | IV | NO | M1b | 2 | bone, lung |
|  | Plasma |  | EGFR: wild type | − | 4865 | EGFR DEL: wild type |  |  |  |  |  |  |
| L12 | Biopsy | 10% | EGFR: DEL exon 19 | EGFR: p.E746_A750del (c.2236_2250del15) (5,1%); | 2143 |  | 74 | IV | NO | M1b | 1 | bone |
|  | Plasma |  | EGFR: DEL exon 19 | EGFR: p.E746_A750delELREA (c.2235_2249del15) (5,97%) | 5227 |  |  |  |  |  |  |  |
| L13 | Biopsy | 60% | EGFR: DEL exon 19 | KRAS: p.G13C (c.37G>T) (10,1%); EGFR: p.E746_A750delELREA (c.2236_2250del15) (4,2%) | 1896 |  | 62 | IV | NO | M1b | 2 | bone and brain |
|  | Plasma |  | EGFR: wild type | EGFR: p.E746_A750delELREA (c.2236_2250del15) (1,99%) | 5174 |  |  |  |  |  |  |  |
| L14 | Biopsy | 80% | EGFR: Ins Ex 20 | EGFR: p.V769_D770insASV (c.2307_2308insGCCAGCGTG) (52,8%) | 2232 |  | 68 | IV | YES | M1b | > 2 | lung, limph nodes, liver, bone |
|  | Plasma |  | EGFR: Ins Ex 20 | EGFR: p.V769_D770insASV (c.2307_2308 ins GCCAGCGTG) (9,2%) | 5369 |  |  |  |  |  |  |  |
| L15 | Cytology | 150 cells | EGFR: c.2573T>G (p.L858R) | EGFR: p.E709K (c.2125G>A) (18,1%); p.L858R (c.2573T>G) (16,2%); | 1987 |  | 74 | IV | NO | M1a | 2 | lung and pleura |
|  | Plasma |  | EGFR: c.2573T>G (p.L858R) | EGFR: p.L858R (c.2573T>G) (7,3%); p.E709K (c.2125G>A) (3,9%) | 5124 |  |  |  |  |  |  |  |
| L16 | Biopsy | 70% | EGFR: c.2573T>G (p.L858R) and Del exon 19 | KRAS: p.G12C (c.34G>T) (3,3%); EGFR: p.E746_A750del (c.2236_2250del15) (12,8%); p.L858R (c.2573T>G) (16,9%) | 2123 | KRAS: p.G12C (c.34G>T) (2,1%) | 54 | IV | YES | M1a | 1 | pleura |
|  | Plasma |  | EGFR: c.2573T>G (p.L858R) | EGFR: p.L858R (c.2573T>G) (5,3%); | 5263 | EGFR DEL: WT |  |  |  |  |  |  |
| L17 | Cytology | 200 cells | EGFR: DEL exon 19 | EGFR: p.E746_S752>V (c.2237_2255>T (50%); TP53: p.R158L (c.473G>T) (18,4%) | 2150 |  | 51 | IV | NO | M1b | 2 | bone and lung |
|  | Plasma |  | EGFR: DEL exon 19 | EGFR: p.E746_S752>V c.2237-2255>T (57,7%); TP53: p.R158L c.473G>T (12,1%) | 4985 |  |  |  |  |  |  |  |
| L18 | Cytology | 200 cells | EGFR: DEL exon 19 | EGFR: p.E746_A750del (c2235_2249 del 15) (19,9%) | 1943 |  | 75 | IV | NO | M1b | 1 | bone |
|  | Plasma |  | EGFR: wild type | − | 5003 |  |  |  |  |  |  |  |
| L19 | Cytology | 500 cells | EGFR: c.2235-2249del15bp; (p.E746-A750del); c.2369C>T (p.T790M) | EGFR: p.E746_A750del (c2235_2249 del 15) (87,7%); p.T790M (c.2369C>T) (4,1%) | 2070 |  | 74 | IV | NO | M1b | > 2 | lung, bone, brain |
|  | Plasma |  | EGFR: DEL exon 19 and T790M | EGFR: p.E746_A750delELREA (c.2235_2249del15) (35,6%); c.2369C>T p.T790M (3,2%); | 5010 |  |  |  |  |  |  |  |
| L20 | Cytology | 700 cells | EGFR: c.2237_2255>T; (p.E746_S752>V) | EGFR: p.E746_S752>V (c.2237_2255>T) (51,3%) | 1980 |  | 55 | IV | NO | M1a | 1 | lung |
|  | Plasma |  | EGFR: wild type | BRAF: p.F595L c.1785T>A (2,1%) | 5232 |  |  |  |  |  |  |  |
| L21 | Cytology | 150 cells | EGFR: Ins Ex 20 | EGFR: p.D770_N771insG c.2310_2311insGGT (35,6%); TP53: p.R248Q c.743G>A (15,2%) | 2007 |  | 61 | IV | NO | M1b | > 2 | bone, brain, limph nodes, liver |
|  | Plasma |  | EGFR: Ins Ex 20 | EGFR: p.D770_N771insG c.2310_2311insGGT (23,7%); TP53: p.R248Q c.743G>A (8,8%) | 5180 |  |  |  |  |  |  |  |
| L22 | Biopsy | 10% | EGFR: c.2573T>G (p.L858R) | EGFR: p.L858R c.2573T>G (6,3%) | 1995 |  | 68 | IV | NO | M1b | > 2 | lymph nodes, lung, pleura |
|  | Plasma |  | EGFR: c.2573T>G (p.L858R) | EGFR: p.L858R c.2573T>G (4,1%) | 4970 |  |  |  |  |  |  |  |
