# Supplementary material for: Limits and potential of targeted sequencing analysis of liquid biopsy in patients with lung and colon carcinoma
Source: Oncotarget. 2016 Jul 19;7(41):66595–605. doi: 10.18632/oncotarget.10704 (PMC5341823; doi:10.18632/oncotarget.10704)
Supplement: Supplementary file 3 [file oncotarget-07-66595-s003.docx]

Supplementary Table S2: - EGFR wild type NSCLC

| DM CROM | Sample type | % or N. neoplastic cells | Expected variants | Mutations found by ION | Coverage | Selected mutations analysed by ddPCR | Age | Stage | Primary tumor resected or not resected | Metastasis (M1a, M1b) | N° metastatic site | Metastatic site |
| --- | --- | --- | --- | --- | --- | --- | --- | --- | --- | --- | --- | --- |
| L23 | Biopsy | 60% | EGFR: wild type | − | 2148 |  | 69 | IV | YES | M1b | 1 | lymph node |
|  | Plasma |  | EGFR: wild type | − | 5487 |  |  |  |  |  |  |  |
| L24 | Biopsy | 60% | EGFR: wild type; KRAS: p.G12D (c.35G>A) | KRAS: p.G12D (c.35G>A) (2,2%); TP53: p.H193R (c.578A>G) (10,4%) | 2325 |  | 64 | IV | YES | M1b | 1 | adrenal gland |
|  | Plasma |  | EGFR: wild type | − | 4999 |  |  |  |  |  |  |  |
| L25 | Biopsy | 90% | EGFR: wild type; KRAS: p.G12C (c.34G>T) | KRAS: p.G12C (c.34G>T)(8,4%) | 2264 |  | 45 | IV | YES | M1b | 2 | brain and lung |
|  | Plasma |  | EGFR: wild type | TP53: p.R248Q; (c.743G>A) (2,3%) | 5007 |  |  |  |  |  |  |  |
| L26 | Biopsy | 80% | EGFR: wild type | − | 2165 |  | 59 | IV | YES | M1b | 1 | brain |
|  | Plasma |  | EGFR: wild type | − | 5078 |  |  |  |  |  |  |  |
| L27 | Biopsy | 60% | EGFR: wild type; KRAS: p.G12V (c.35G>T) | KRAS: p.G12V (c.35G>T) (2,3%); TP53: p.R273H (c.818G>A) (7,4%) | 2205 |  | 67 | IV | NO | M1b | >2 | bone, lymph nodes, pleura |
|  | Plasma |  | EGFR: wild type | − | 5126 |  |  |  |  |  |  |  |
| L28 | Biopsy | 5% | EGFR: wild type | TP53: p.R273L (c.818G>T) (5,1%) | 1998 |  | 55 | IV | NO | M1b | >2 | bone, brain, liver, limph nodes |
|  | Plasma |  | EGFR: wild type; KRAS: p.G12A (c.35G>C) | KRAS: p.G12A (c.35G>C) (4,5%) | 5230 | KRAS G12A: G12A |  |  |  |  |  |  |
| L29 | Cytology | 40 cells | EGFR: wild type | − | 2070 | EGFR DEL: DEL (0.23%) | 76 | IV | NO | M1b | >2 | brain, liver, adrenal gland |
|  | Plasma |  | EGFR: wild type | EGFR: p.E746_A750del (c.2235_2249del15) (3,4%); | 5180 | EGFR: Del (4%) |  |  |  |  |  |  |
| L30 | Cytology | 350 cells | EGFR: wild type | − | 2125 |  | 63 | IV | NO | M1b | 2 | bone and liver |
|  | Plasma |  | EGFR: wild type | − | 4996 |  |  |  |  |  |  |  |
| L31 | Cytology | 300 cells | EGFR: wild type | MET: p.T1010I c.3029C>T (42,2%); KRAS: p.Q61H (c.183A>T) (79,8%); TP53: p.R248Q (c.743G>A) (48,5%) | 1987 |  | 63 | IV | NO | M1a | 1 | lung |
|  | Plasma |  | EGFR: wild type | MET: p.T1010I c.3029C>T (48,1%); PIK3CA: p.M1043V (c.3127A>G) (2,6%) | 5002 |  |  |  |  |  |  |  |
| L32 | Biopsy | 70% | EGFR: wild type | KRAS: p.Q61L (c.182A>T) (10%); TP53: p.R248L c.743G>T (11,8%) | 2236 |  | 48 | IV | YES | M1b | 2 | brain and adrenal gland |
|  | Plasma |  | EGFR: wild type | TP53: p.Y220C c.659A>G (2,1%) | 4874 |  |  |  |  |  |  |  |
| L33 | Biopsy | 80% | EGFR: wild type | CTNNB1: p.S37C (c.110C>G)(13,3%); | 2215 | EGFR DEL: DEL (0.76%) | 58 | IV | NO | M1b | 1 | brain |
|  | Plasma |  | EGFR: wild type | EGFR: p.E746_A750Del15 (c.2236_2250Del15) (1,6%); CTNNB1: p.S37C (c.110C>G)(12,3%); | 5007 | EGFR: Del (0,8%) |  |  |  |  |  |  |
| L34 | Cytology | 150 cells | EGFR: wild type; KRAS: p.G12C (c.34G>T ) | KRAS: p.G12C (c.34G>T ) (27,4%) | 1981 |  | 57 | IV | NO | M1b | 2 | bone and lung |
|  | Plasma |  | EGFR: wild type | KRAS: p.G12C (c.34G>T ) (11,1%) | 4864 |  |  |  |  |  |  |  |
| L35 | Biopsy | 70% | EGFR: wild type | − | 2205 |  | 55 | IV | YES | M1b | >2 | bone, lung, lymph nodes |
|  | Plasma |  | EGFR: wild type | − | 5231 |  |  |  |  |  |  |  |
| L36 | Biopsy | 80% | EGFR: wild type | − | 2147 |  | 68 | IV | NO | M1b | >2 | bone, liver, adrenal gland |
|  | Plasma |  | EGFR: wild type | − | 5095 |  |  |  |  |  |  |  |
| L37 | Cytology | 100 cells | EGFR: wild type | TP53: p.F270S c.809T>C (59,3%) | 2225 |  | 52 | IV | NO | M1b | 2 | adrenal gland and lymph nodes |
|  | Plasma |  | EGFR: wild type | TP53: p.F270S c.809T>C (30,9%) | 5315 |  |  |  |  |  |  |  |
| L38 | Biopsy | 90% | EGFR: wild type; KRAS: p.G12D (c.35G>A) | KRAS: p.G12D (c.35G>A) (31,2%); CTNNB1: p.S37F (c.110C>T) (4,7%) | 2435 |  | 48 | IV | NO | M1b | 1 | bone |
|  | Plasma |  | EGFR: wild type | − | 5190 |  |  |  |  |  |  |  |
| L39 | Cytology | 150 cells | EGFR: wild type | − | 1980 |  | 62 | IV | NO | M1b | 2 | bone and lymph nodes |
|  | Plasma |  | EGFR: wild type | − | 4875 |  |  |  |  |  |  |  |
| L40 | Biopsy | 80% | EGFR: wild type | − | 2005 |  | 64 | IV | NO | M1b | 2 | brain and soft tissue |
|  | Plasma |  | EGFR: wild type | − | 5123 |  |  |  |  |  |  |  |
| L41 | Biopsy | 70% | EGFR: wild type | − | 2124 | KRAS G13D: G13D (0,03%) | 63 | IV | YES | M1b | 2 | spleen and lymp nodes |
|  | Plasma |  | EGFR: wild type; KRAS: p.G13D (c.38G>A) | KRAS: p.G13D (c.38G>A) (5,6%) | 5037 |  |  |  |  |  |  |  |
| L42 | Biopsy | 60% | EGFR: wild type | KRAS: p.G12D (c.35G>A) (11,3%); TP53: p.S99F (c.296C>T) (7,8%) | 2225 |  | 75 | IV | YES | M1a | 1 | lung |
|  | Plasma |  | EGFR: wild type | − | 5246 |  |  |  |  |  |  |  |
| L43 | Biopsy | 60% | EGFR: wild type | − | 2365 |  | 76 | IV | NO | M1b | 2 | liver and brain |
|  | Plasma |  | EGFR: wild type | − | 5148 |  |  |  |  |  |  |  |
| L44 | Biopsy | 90% | EGFR: wild type | − | 1987 |  | 65 | IV | NO | M1b | 1 | bone |
|  | Plasma |  | EGFR: wild type | − | 5207 |  |  |  |  |  |  |  |
