# Supplementary material for: Limits and potential of targeted sequencing analysis of liquid biopsy in patients with lung and colon carcinoma
Source: Oncotarget. 2016 Jul 19;7(41):66595–605. doi: 10.18632/oncotarget.10704 (PMC5341823; doi:10.18632/oncotarget.10704)
Supplement: Supplementary file 4 [file oncotarget-07-66595-s004.docx]

Supplementary Table S3: - RAS mutant CRC

| ID | Sample type | % tumor cells | Expected variants  (KRAS, NRAS) | Mutations found by ION | Coverage | Selected mutations analysed by ddPCR | Age | Stage | Primary tumor resected or not resected | N° metastatic sites | Metastatic site |
| --- | --- | --- | --- | --- | --- | --- | --- | --- | --- | --- | --- |
| C1 | Tissue | 50 | NRAS: p.Q61R (c.182A>G) | NRAS: p.Q61R (c.182A>G) (20%) | 1980 |  | 41 | IV | NO | 1 | liver |
|  | Plasma |  |  | NRAS: p.Q61R (c.182A>G) (41,2%) | 4550 |  |  |  |  |  |  |
| C2 | Tissue | 80 | KRAS: p.G12C (c.34G>T) | KRAS: p.G12C (c.34G>T) (19,5%); TP53: p.R175H (c.524G>A) (18,8%) | 2020 |  | 58 | IV | YES | > 2 | liver, peritoneum, bone |
|  | Plasma |  |  | KRAS: p.G12V (c.35G>T)(1,7%)* | 4874 | KRAS: p.G12V (c.35G>T) (0.6%); KRAS G12C: wild type |  |  |  |  |  |
| C3 | Tissue | 80 | KRAS: p.G12D (c.35G>A) | KRAS: p.G12D (c.35G>A) (13,2%); PIK3CA: p.E545K (c.1633G>A) (9,5%) | 2110 |  | 48 | IV | YES | 1 | liver |
|  | Plasma |  |  | KRAS: p.G12D c.35G>A (3,8%); PIK3CA: p.E545K (c.1633G>A) (3,4%) | 4890 |  |  |  |  |  |  |
| C4 | Tissue | 50 | KRAS: p.G12V (c.35G>T) | KRAS: p.G12V (c.35G>T) (42,2%); TP53: p.R273H (c.818G>A) (28,2%) | 1940 |  | 68 | IV | NO | 2 | liver and lypmh nodes |
|  | Plasma |  |  | KRAS: p.G12V (c.35G>T) (6,8%) | 4415 |  |  |  |  |  |  |
| C5 | Tissue | 70 | KRAS: p.G12D (c.35G>A) | KRAS: p.G12D (c.35G>A)(35,5%) | 2430 |  | 76 | IV | YES | 1 | liver |
|  | Plasma |  |  | KRAS: p.G12D (c.35G>A) (8,6%) | 4985 |  |  |  |  |  |  |
| C6 | Tissue | 80 | KRAS: p.G12V(c.35G>T) | KRAS: p.G12V(c.35G>T)(39,4%); PIK3CA: p.G1049S(c.3145G>A ) (4,6%); SMAD4: p.R497H(c.1490G>A (13,3%); TP53: p.R282W(c.844C>T)(6,7%) | 2185 |  | 61 | IV | YES | 2 | lung, lymph nodes |
|  | Plasma |  |  | KRAS: p.G12V(c.35G>T)(7,7%); | 4755 |  |  |  |  |  |  |
| C7 | Tissue | 80 | NRAS: p.Q61R(c.182A>G) | NRAS: p.Q61R(c.182A>G)(53,4%) | 2230 |  | 62 | IV | YES | 2 | liver and peritoneum |
|  | Plasma |  |  | − | 5072 |  |  |  |  |  |  |
| C8 | Tissue | 70 | KRAS: p.G13D (c.G38A) | KRAS: p.G13D(c.G38A)(35,5%); TP53: p.R175H(c.524G>A) (57,5%) | 2270 |  | 55 | IV | YES | 2 | lung and lymph nodes |
|  | Plasma |  |  | KRAS: p.G13D(c.G38A)(16,8%); TP53: p.R175H(c.524G>A) (30,7%) | 4990 |  |  |  |  |  |  |
| C9 | Tissue | 50 | KRAS: p.G12S (c.34G>A) | KRAS: p.G12S (c.34G>A) (39,5%); PIK3CA: p.H1047R (c.3140A>G) (24,5%) | 2125 |  | 74 | IV | YES | 2 | lung and liver |
|  | Plasma |  |  | KRAS: p.G12S (c.34G>A)(2,8%)*; PIK3CA: p.H1047R;(c.3140A>G)(1,9%)* | 5248 |  |  |  |  |  |  |
| C10 | Tissue | 60 | KRAS: p.V14I (c.40G>A) | KRAS: p.V14I (c.40G>A) (17%) | 2170 |  | 54 | IV | YES | 1 | peritoneum |
|  | Plasma |  |  | KRAS: p.V14I (c.40G>A) (5,24%) | 5123 |  |  |  |  |  |  |
| C11 | Tissue | 60 | KRAS: p.G12D (c.35G>A) | KRAS: p.G12D (c.35G>A) (14,7%); PIK3CA: p.E545K (c.1633G>A) (13,5%) | 2056 |  | 74 | IV | NO | 1 | liver |
|  | Plasma |  |  | KRAS: p.G12D (c.35G>A) (6,7%); PIK3CA: p.E545K (c.1633G>A) (14,1%) | 4896 |  |  |  |  |  |  |
| C12 | Tissue | 70 | KRAS: p.G12S (c.34G>A) | KRAS: p.G12S (c.34G>A) (51,9%) | 2230 |  | 67 | IV | YES | 1 | liver |
|  | Plasma |  |  | KRAS: p.G12S (c.34G>A) (16%) | 5015 |  |  |  |  |  |  |
| C13 | Tissue | 90 | KRAS: p.A146T (c.436G>A) | KRAS: p.A146T (c.436G>A) (27,2%); TP53: p.R175H (c.524G>A) (54%) | 2149 |  | 67 | IV | YES | > 2 | lung, peritoneum, lymph nodes |
|  | Plasma |  |  | − | 4988 | KRAS: p.A146T (c.436G>A) (0.93%) |  |  |  |  |  |
| C14 | Tissue | 40 | KRAS: p.A59E (c.176C>A) | KRAS: p.A59E (c.176C>A) (25,6%); BRAF: p.G466E (c.1397G>A) (22,9%) | 2075 |  | 54 | IV | NO | > 2 | liver, lung, peritoneum |
|  | Plasma |  |  | KRAS: p.A59E (c.176C>A) (12,1%); BRAF: p.G466E(c.1397G>A)(16,6%) | 4998 |  |  |  |  |  |  |
| C15 | Tissue | 30 | KRAS: p.G12D(c.35G>A) | KRAS: p.G12D(c.35G>A)(7,9%) | 2158 |  | 77 | IV | YES | 1 | lung |
|  | Plasma |  |  | − | 4987 | KRAS G12D: wild type |  |  |  |  |  |
| C16 | Tissue | 60 | KRAS: p.G12D(c.35G>A) | KRAS: p.G12D(c.35G>A)(23,5%) | 2143 |  | 35 | IV | NO | 1 | liver |
|  | Plasma |  |  | KRAS: p.G12D(c.35G>A)(38,3%) | 4978 |  |  |  |  |  |  |
| C17 | Tissue | 70 | KRAS: p.G12D(c.35G>A) | KRAS: p.G12D(c.35G>A)(55,6%) | 2126 |  | 75 | IV | YES | > 2 | lung, lymph nodes, bone |
|  | Plasma |  |  | KRAS: p.G12D(c.35G>A)(20,5%) | 5005 |  |  |  |  |  |  |
| C18 | Tissue | 80 | KRAS: p.G12S (c.34G>A) | KRAS: p.G12S (c.34G>A) (23,1%); PIK3CA: p.H1047R (c.3140A>G)(13,6%) | 2124 |  | 56 | IV | YES | 1 | peritoneum |
|  | Plasma |  |  | PIK3CA: p.H1047R (c.3140A>G)(2,0%) | 5123 | KRAS: p.G12S (c.34G>A) (0.84%) |  |  |  |  |  |
| C19 | Tissue | 80 | KRAS: p.G12V(c.35G>T) | KRAS:p.G12V(c.35G>T)(39,7%); SMAD4: p.G358(c.1072G>T)(43,4%); TP53: p.R248W(c.742C>T)(42,2%) | 2078 |  | 58 | IV | YES | 1 | lung |
|  | Plasma |  |  | − | 4995 | KRAS:p.G12V (c.35G>T) (0.5%) |  |  |  |  |  |
| C20 | Tissue | 70 | KRAS, NRAS: wild type | EGFR: p.G719S (c.2155G>A) (19,4%) | 2080 |  | 61 | IV | YES | > 2 | liver, lung, lymph nodes |
|  | Plasma |  |  | − | 5189 |  |  |  |  |  |  |
| C21 | Tissue | 80 | KRAS, NRAS: wild type | − | 2240 |  | 70 | IV | YES | 1 | peritoneum |
|  | Plasma |  |  | − | 5230 |  |  |  |  |  |  |
| C22 | Tissue | 60 | KRAS, NRAS: wild type | TP53: p.R248Q (c.743G>A) (4,1%) | 2060 |  | 65 | IV | NO | > 2 | lymph nodes, peritoneum, bone |
|  | Plasma |  |  | TP53: p.R248Q (c.743G>A) (3,5%) | 4995 |  |  |  |  |  |  |
| C23 | Tissue | 60 | KRAS, NRAS: wild type | PIK3CA: p.Q546P (c.1637A>C) (2,9%); TP53: p.R273C (c.817C>T) (7,5%)* | 2120 |  | 60 | IV | NO | > 2 | liver, lymph nodes, peritoneum |
|  | Plasma |  |  | PIK3CA: p.Q546P (c.1637A>C) (46,4%); TP53: p.R273C (c.817C>T) (83%)* | 5005 |  |  |  |  |  |  |
| C24 | Tissue | 70 | KRAS, NRAS: wild type | TP53: p.R175H(c.524G>A) (60,9%) | 1945 |  | 74 | IV | NO | > 2 | liver, lung, lymph nodes |
|  | Plasma |  |  | TP53: p.R175H(c.524G>A) (76,5%) | 4765 |  |  |  |  |  |  |
| C25 | Tissue | 40 | KRAS, NRAS: wild type | BRAF: p.V600E(c.1799T>A)(10%); SMAD4: p.D351H(c.1051G>C)(12,4%) | 2180 |  | 72 | IV | NO | > 2 | liver, lung, lymph nodes, pleura, peritoneum |
|  | Plasma |  |  | BRAF: p.V600E(c.1799T>A)(50%); SMAD4: p.D351H(c.1051G>C)(70,6%) | 5050 |  |  |  |  |  |  |
| C26 | Tissue | 80 | KRAS, NRAS: wild type | − | 2147 |  | 54 | IV | YES | 1 | liver |
|  | Plasma |  |  | TP53: p.R213Q (c.638G>A) (1,8%) | 4983 |  |  |  |  |  |  |
| C27 | Tissue | 80 | KRAS, NRAS: wild type | TP53: p.R248Q (c.743G>A) (28,3%) | 2070 |  | 82 | IV | NO | 2 | liver and lymph nodes |
|  | Plasma |  |  | TP53: p.R248Q (c.743G>A) (69,1%) | 5010 |  |  |  |  |  |  |
| C28 | Tissue | 50 | KRAS, NRAS: wild type | TP53: p.R248Q (c.743G>A) (25,1%) | 1997 |  | 67 | IV | YES | 1 | lung |
|  | Plasma |  |  | − | 4996 |  |  |  |  |  |  |
| C29 | Tissue | 30 | KRAS, NRAS: wild type | EGFR: p.S768I (c.2303G>T) (1,9%) | 2247 |  | 67 | IV | NO | > 2 | liver, lung, peritoneum |
|  | Plasma |  |  | − | 5362 |  |  |  |  |  |  |
| C30 | Tissue | 40 | KRAS, NRAS: wild type | − | 2110 |  | 60 | IV | NO | > 2 | liver, peritoneum, lymph nodes |
|  | Plasma |  |  | − | 5023 |  |  |  |  |  |  |
| C31 | Tissue | 50 | KRAS, NRAS: wild type | BRAF: p.V600E (c.1799T>A) (6,8%) | 2223 |  | 74 | IV | NO | 1 | liver |
|  | Plasma |  |  | BRAF: p.V600E(c.1799T>A)(41%); | 5225 |  |  |  |  |  |  |
| C32 | Tissue | 80 | KRAS, NRAS: wild type | − | 1995 |  | 45 | IV | YES | 2 | liver and bone |
|  | Plasma |  |  | − | 4996 |  |  |  |  |  |  |
| C33 | Tissue | 10 | KRAS, NRAS: wild type | − | 2236 |  | 48 | IV | NO | 2 | liver and lung |
|  | Plasma |  |  | − | 5170 |  |  |  |  |  |  |
| C34 | Tissue | 80 | KRAS, NRAS: wild type | FBXW7: p.R465C(c.1393C>T)(67,9%) | 1995 |  | 79 | IV | NO | 2 | lung and peritoneum |
|  | Plasma |  |  | FBXW7: p.R465C(c.1393C>T)(15,3%) | 4987 |  |  |  |  |  |  |
| C35 | Tissue | 20 | KRAS, NRAS: wild type | PIK3CA: p.T1025A (c.3073A>G)(10,9%) | 2027 |  | 49 | IV | NO | 1 | liver |
|  | Plasma |  |  | − | 4869 |  |  |  |  |  |  |

**variant detected with the new design of the panel.
